# Buprenorphine versus dihydrocodeine for opiate detoxification in primary care: a randomised controlled trial

**DOI:** 10.1186/1471-2296-8-3

**Published:** 2007-01-08

**Authors:** Nat MJ Wright, Laura Sheard, Charlotte NE Tompkins, Clive E Adams, Victoria L Allgar, Nicola S Oldham

**Affiliations:** 1 Centre for Research in Primary Care, 71-75 Clarendon Road, Leeds, LS2 9PL, UK; 2 Department of Psychiatry, 15 Hyde Terrace, Leeds, LS2 9L, UK; 3 Formerly of NFA Health Centre for Homeless People, 68 York Street, Leeds, LS9 8AA, UK

## Abstract

**Background:**

Many drug users present to primary care requesting detoxification from illicit opiates. There are a number of detoxification agents but no recommended drug of choice. The purpose of this study is to compare buprenorphine with dihydrocodeine for detoxification from illicit opiates in primary care.

**Methods:**

Open label randomised controlled trial in NHS Primary Care (General Practices), Leeds, UK. Sixty consenting adults using illicit opiates received either daily sublingual buprenorphine or daily oral dihydrocodeine. Reducing regimens for both interventions were at the discretion of prescribing doctor within a standard regimen of not more than 15 days. Primary outcome was abstinence from illicit opiates at final prescription as indicated by a urine sample. Secondary outcomes during detoxification period and at three and six months post detoxification were recorded.

**Results:**

Only 23% completed the prescribed course of detoxification medication and gave a urine sample on collection of their final prescription. Risk of non-completion of detoxification was reduced if allocated buprenorphine (68% vs 88%, RR 0.58 CI 0.35–0.96, p = 0.065). A higher proportion of people allocated to buprenorphine provided a clean urine sample compared with those who received dihydrocodeine (21% vs 3%, RR 2.06 CI 1.33–3.21, p = 0.028). People allocated to buprenorphine had fewer visits to professional carers during detoxification and more were abstinent at three months (10 vs 4, RR 1.55 CI 0.96–2.52) and six months post detoxification (7 vs 3, RR 1.45 CI 0.84–2.49).

**Conclusion:**

Informative randomised trials evaluating routine care within the primary care setting are possible amongst drug using populations. This small study generates unique data on commonly used treatment regimens.

## Background

In the United Kingdom (UK), policy directives have highlighted the importance of offering either maintenance or detoxification to illicit opiate users within an agreed plan of care [1]. This is in response to routine practice by some treatment providers of 'gradual reduction' of opiate maintenance treatment – a regimen without a supporting evidence base [2]. Opiate detoxification, using one of various therapeutic agents, remains an important part of drug management for some illicit opiate users. However, neither the evidence base nor UK national guidelines recommend a 'drug of choice' [1]. Understandably, there has been a call for randomised controlled trials (RCTs) in this area [3]. In primary care, methadone is commonly used, with reductions in the dose over 7–21 days [4]. Methadone has a long half life [5,6] and patients often report distressing withdrawal symptoms in the latter stages of detoxification [6]. This has meant increasing use of alternative agents such as clonidine, lofexidine, dihydrocodeine and, more recently, buprenorphine. The hypotensive effects of clonidine [4] have make it unacceptable for use in primary care and the reduced ability of lofexidine to control withdrawal, coupled with its high cost have resulted in limited clinical uptake [4].

The use of sublingual buprenorphine is relatively new in the UK for opiate detoxification and there have been only two randomised controlled trials (RCTs) comparing it with methadone for this purpose [7,8]. Buprenorphine has been more commonly used as a drug of comparison in trials of opiate *maintenance *[9-16]. A recent Cochrane review assessing methadone and buprenorphine for the management of opioid withdrawal found no significant difference between these two agents [17]. In this context, buprenorphine has a good safety profile, better retention in treatment and lower withdrawal severity [18-22]. Sublingual buprenorphine is increasingly being prescribed by General Practitioners (GPs) for opiate *detoxification *[23] despite limited clinical and research evidence.

Dihydrocodeine has a shorter half life than methadone and has been widely used in both primary care and prison drug treatment settings for opiate detoxification. Whilst some commentators have documented success with dihydrocodeine [24,25] others have expressed concerns regarding its effects, particularly the potential diversion into the street economy [26]. Despite routine use, dihydrocodeine has rarely been studied for the purposes of opiate detoxification [24] but has been compared in a randomised controlled trial with buprenorphine for postoperative pain [27].

When comparing methadone, dihydrocodeine and buprenorphine it is important to note several factors which may impact upon prescribing and use of these agents. Dihydrocodeine is cheaper than methadone and both methadone and dihydrocodeine are substantially cheaper than buprenorphine. The latter has been subject to heavy pharmaceutical marketing. Buprenorphine and dihydrocodeine have a better safety profile than methadone, which has a high toxicity which (rarely) can result in death [28]. All three agents have the potential for street diversion but dihydrocodeine is the hardest to control, with consumption usually being unsupervised. Methadone is the easiest to manage and buprenorphine seems to be somewhere in-between.

The care of people using illicit opiates has changed over recent years. Strang et al (2005) [29] surveyed GPs and found that half had seen at least one opiate user in a four week period, compared to only 19% in a 1986 survey [30]. There has been a significant increase in the number of GPs becoming involved in the care of drug users [31]. Consequently, many short term opiate detoxifications are now undertaken in primary care. The absence of robust evidence underpinning many of the clinical decisions made within primary care has already been highlighted [32]. LEEDS (Leeds Evaluation of Efficacy of Detoxification Study) is a response to this challenge and compared dihydrocodeine with buprenorphine for opiate detoxification within the UK primary care setting.

## Methods

### Design and setting

LEEDS was conducted in ten general practices (6 of which randomised participants) in Leeds, UK (population ~750,000). We used a randomised controlled trial design to compare open giving of oral dihydrocodeine tartrate with open giving of sublingual buprenorphine. Randomisation was by random block size, stratified by practice, using Microsoft Excel RAND function. This was undertaken by the Department of Psychiatry, University of Leeds, and was concealed from clinicians prescribing interventions. The name of the allocated intervention was obscured within fully opaque sealed envelopes [33]. All envelopes were opened in strict order, confirmed by an investigator independent of the clinical interface. The outside of the randomisation envelope contained a brief information form to be completed which requested the patient's practice number, date of birth, contact telephone number and date of first prescription. Two questions also served to rate the severity of addiction of the participant from the view of the GP. Once the GP/drug worker opened the LEEDS envelope both practitioner and patient knew the intervention, standard clinical care resumed and the patient made subsequent appointments with the GP and/or drugs worker. To detect with 80% power a difference in treatment effectiveness of 25% between groups (50% versus 25%) at 5% level of significance, it was calculated that 120 participants would be needed in the study. However within the time frame for recruitment we were only able to recruit 60 participants. Randomisation took place between August 2002 and May 2004. Full methods are reported elsewhere [33].

The Leeds Teaching Hospitals Local NHS Research Ethics Committee (LREC) approved the study in April 2002. Informed written consent was obtained from each patient following receipt of a participant information leaflet prior to their involvement in the trial.

### Interventions

Buprenorphine was prescribed on an FP10 MDA prescription. This allows daily dispensing under supervision of a pharmacist. Daily supervised administration of dihydrocodeine tablets is not possible in the UK as it cannot be prescribed on FP10 MDA prescriptions. As such, buprenorphine was dispensed either as 8 mg, 2 mg or 0.4 mg sublingual tablet preparation under daily supervision. Dihydrocodeine was dispensed as 30 mg rapid release tablet preparation in take home installments. Each installment was for a minimum of three and a maximum of 4 daily doses. The reducing regimens for both interventions were at the discretion of the prescribing doctor and within the standard regimen which was approximately 15 days (Tables 1 and 2). However, clinicians were free to titrate doses against withdrawal symptoms. What was being randomised was the *open giving *of the drugs even if that meant that participants were not given in the opinion of the prescribing doctor pharmacologically equivalent dosages.

**Table 1:** Standard buprenorphine detoxification

**Day**	**Dose (mg)**
1	6
2	8
3	8
4	6
5	6
6	4
7	3.6
8	3.2
9	2.8
10	2.4
11	2.0
12	1.6
13	1.2
14	0.8
15	0.4

**Table 2:** Standard dihydrocodeine detoxification

Day	Number of 30 mg tablets	Morning	Midday	Evening	Night-time
1	18	5	4	4	5
2	20	5	5	5	5
3	18	5	4	4	5
4	16	4	4	4	4
5	14	4	3	3	4
6	12	3	3	3	3
7	10	3	2	2	3
8	9	2	2	2	3
9	8	2	2	2	2
10	7	2	1	2	2
11	6	2	1	1	2
12	5	1	1	1	2
13	4	1	1	1	1
14	3	1	1		1
15	2	1			1

### Inclusion and exclusion criteria

Patients were eligible for the study if they were: aged 18 years or over, using street opiates as confirmed by a urine sample taken at first assessment, wishing to detoxify through the standard monitored process, willing to remain abstinent from opiates and to give informed consent. Patients were excluded if they had contra-indications to dihydrocodeine or buprenorphine or had been randomised into the trial previously.

### Outcomes

The primary outcome was abstinence from illicit heroin at final prescription, as indicated by urine test. *A priori *we classed unsuccessful detoxification as: the final urine tested positive for metabolic breakdown products of heroin (morphine or 6-mono-acetyl morphine); urine tested positive for opiates commonly found in street heroin (codeine); the patient did not provide a final urine sample; did not finish detoxification or reported using street opiates during the period of detoxification. We recorded the secondary outcomes of inappropriate use of prescribed medication, overdose and admission to hospital or Accident and Emergency (A&E) and number of GP/drug worker visits during the detoxification period. At three and six month post detoxification, follow up data were recorded. These outcomes were: whether the person was still alive, abstinent from opiates, in receipt of sickness certification and their pattern of service use.

### Statistical analysis

Outcome data were analysed using Epi Info v 3.3.2 and SPSS software with relative risk tests for categorical data and unpaired t-tests for continuous data.

## Results

**Figure 1:**
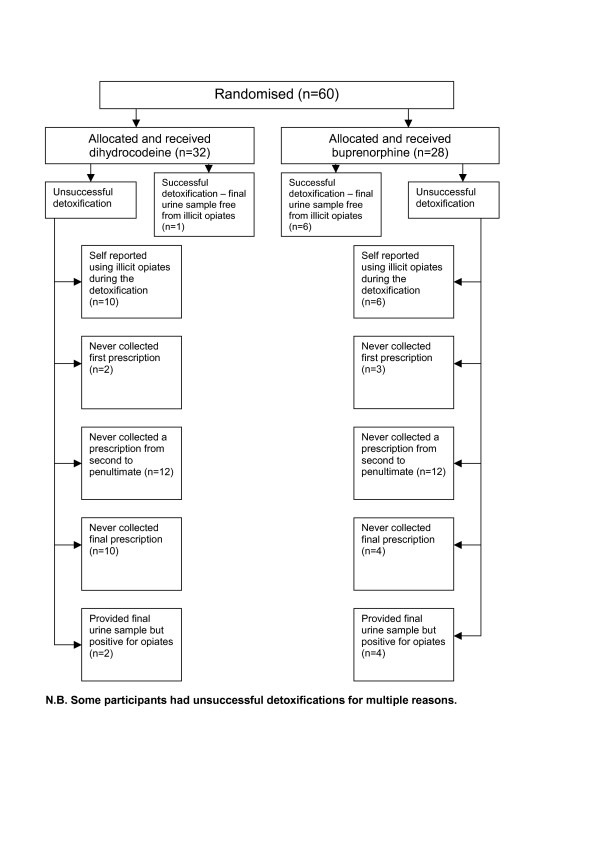


Sixty people using illicit opiates took part in LEEDS (Figure 1). This comprised of 42 men and 18 women, with an average age of 28 years. 58% were homeless or unstably housed. There were no significant differences for those allocated to one regimen or the other (Table 3).

**Table 3:** Demographic characteristics and prognostic factors

	**Buprenorphine (total 28)**			**Dihydrocodeine (total 32)**		
**Age **mean (SD)	29.9 (5.1)			29.0 (7.3)		
**Sex**	19 M 9 F			23 M 9 F		
**Pattern of use**						
**How are opiates taken?**						
IV	14 (50%)			24 (75%)		
Smoking	13 (46%)			8 (25%)		
Both	1 (4%)			0		
**Current daily use (minimum) (£) **mean	17.1 (8.1)			15.6 (7.2)		
**Current daily use maximum (£) **mean	23.2 (12.1)			18.1 (9.0)		
**Duration taking opiates (years) **mean	8.8 (4.9)			7.0 (3.7)		
**Illicit opiates in initial urine**	23 (82%)			27 (84%)		
**Other drugs in initial urine**	18 (64%)			12 (37%)		
**Prognostic factors**						
**'Severely dependent**'	8 (28%)			10 (31%)		
**GP's prediction of whether would be off opiates by end of detox**	**Definitely not**	**Not sure**	**Very sure**	**Definitely not**	**Not sure**	**Very sure**
	0	22 (78%)	6 (21%)	2 (6%)	22 (69%)	8 (25%)
	**Yes**	**No**	**D/K**	**Yes**	**No**	**D/K**
**Previous detoxes?**	24 (87%)	3 (11%)	1 (4%)	25 (78%)	6 (19%)	1 (3%)
**Successful detoxes?**	15 (63%)	9 (38%)	0	15 (60%)	9 (36%)	1 (4%)
**Employed?**	4 (14%)	19 (68%)	5 (18%)	4 (13%)	19 (59%)	9 (28%)
**MED-3?**	6 (21%)	8 (29%)	14 (50%)	5 (16%)	13 (41%)	14 (44%)
**Non using friends?**	12 (43%)	5 (18%)	11 (39%)	13 (41%)	3 (9%)	16 (50%)
**Anyone supportive of detox?**	22 (79%)	4 (14%)	2 (7%)	24 (75%)	1 (3%)	7 (22%)

Overall, only 13 people (23%) completed the prescribed course of detoxification medication and gave a urine sample on collection of their final prescription (Table 4). There was an increased chance of completing the prescribing regime if allocated buprenorphine though this finding was of borderline statistical significance (68% vs 88%, RR 0.58 CI 0.35–0.96, p = 0.065). At completion of detoxification, by intention to treat analysis we found a higher proportion of people allocated to buprenorphine provided a urine sample negative for opiates (abstinent) compared with those who received dihydrocodeine (21% vs 3%, RR 2.06 CI 1.33–3.21, p = 0.028). This suggestion of an enhanced therapeutic effect with buprenorphine was negated if we assumed that the proportions of those returning with clean urine per group were representative of those who did not return. Had all the medication been both prescribed and dispensed according to the standard regimes, an expected mean prescribed dose for each dihydrocodeine detoxification would be 4560 mg and 56 mg for each buprenorphine dose. The actual amount of milligrams (mean) and duration of detoxification in days (mean) prescribed for each dihydrocodeine detoxification was 4111 mg (90% of expected dose) over 12 days and 32.9 mg (59% of expected dose) of buprenorphine over 9 days (both rounded to nearest day). This would indicate under-prescribing by doctors for both regimes as 4290 mg (94% of expected dose) would have expected to be prescribed over 12 days for dihydrocodeine and 47.6 mg (85% of expected dose) over 9 days for buprenorphine.

**Table 4:** Results

	**Buprenorphine (total 28)**	**Dihydrocodeine (total 32)**	**Odds Ratio (95% CI)**	**Relative Risk (95% CI)**	**P value**
**By end of detox**					
**Final urine sample**	9 (32%)	4 (13%)	3.32 (0.77–15.22)	1.71 (1.04–2.83)	**0.065**
**Non-ITT Clean urine**	6/9 (67%)	1/4 (25%)	6.00 (0.28–246)	1.71 (0.73–4.03)	**0.164**
ITT-1* clean urine	6/28 (21%)	1/32 (3%)	8.45 (0.89–200)	2.06 (1.33–3.21)	**0.028**
ITT-2* clean urine	6+3/28	1+8/32	1.21 (0.35–4.21)	1.11 (0.63–1.95)	**0.735**
**Leaving early**	19 (68%)	28 (88%)	0.30 (0.07–1.30)	0.58 (0.35–0.96)	**0.065**
**Overdose**	0	0	Not applicable		
**Inappropriate use of allocated drug**	0	0	Not applicable		
**A&E attendance**	0	0	Not applicable		
**Admitted**	0	0	Not applicable		
**Overdose**	0	0	Not applicable		
**GP visits – mean**	2.2 (1.05)	2.8 (1.33)			**0.06**
**Drug worker visits – mean**	0.17 (0.47)	1.31 (0.59)			**0.343**
**At 3 months post detoxification**					
**Abstinent**	10/27 (37%)	4/24 (17%)	2.94 (0.67–13.78)	1.55 (0.96–2.52)	**0.104**
**Dead**	0/27	0/28	Not applicable		
**On Med 3 sick note**	10/16 (63%)	6/14 (43%)	2.22 (0.41–12.65)	1.46 (0.71–2.98)	**0.282**
**A&E attendance**	2/27 (7%)	2/28 (7%)	1.04 (0.09–11.46)	1.06 (0.38–2.94)	**0.970**
**Hospital attendance**	1/27 (4%)	2/28 (7%)	0.5 (0.02–7.75)	0.67 (0.13–3.38)	**0.574**
**GP visits – mean**	5.04 (4.85)	4.61 (4.10)			**0.724**
**Drug worker visits – mean**	1.22 (1.67)	1.14 (1.53)			**0.855**
**At 6 months post detoxification**					
**Abstinent**	7/22 (32%)	3/19 (16%)	2.49 (0.45–15.15)	1.45 (0.84–2.49)	**0.233**
**Dead**	0/23	0/20	Not applicable		
**On Med 3 sick note**	3/11 (27%)	2/11 (18%)	1.69 (0.16–20.05)	1.27 (0.53–3.06)	**0.611**
**A&E attendance**	0/22	0/20	Not applicable		
**Hospital attendance**	0/22	2/20 (10%)	0.16 (0.01–3.64)		**0.129**
**GP visits – mean**	7.54 (6.71)	6.48 (6.07)			**0.582**
**Drug worker visits – mean**	1.74 (2.40)	2.65 (2.89)			**0.265**

• Assumption 1 = everybody not returning for final urine test had not clean urine

• Assumption 2 = everybody not returning for final urine test had same proportion of not clean urine as those who did

There was no statistically significant differences for any other outcomes although throughout the trial people allocated to buprenorphine did better than those on dihydrocodeine. For example people allocated to buprenorphine had fewer visits to the GP and drugs worker during detoxification, and more were abstinent at the three month (10 vs 4, RR 1.55 CI 0.96–2.52) and six month (7 vs 3, RR 1.45 CI 0.84–2.49) follow up. These findings were of borderline statistical significance. No serious adverse events were reported for any participants.

## Discussion

Commentators have listed significant barriers to conducting randomised controlled trials in the primary care setting [34,35]. Barriers certainly may include lack of clinical equipoise towards interventions and patient preference for a particular treatment, [36] as well as logistical problems (principally the busy primary care workplace as not being conducive to practitioner participation) and over-optimism regarding recruitment [37]. This study, however, was at the outset designed collaboratively between primary care and secondary care researchers to be conducted specifically in the primary care setting. It did not greatly complicate routine treatment and recorded clear and concrete outcomes of relevance to the primary care drug treatment field [33]. The LEEDS project team sought GPs experience of being involved in the trial through a cross sectional survey. Details of the practicalities of conducting LEEDS (including recruitment issues and equipoise) have been fully described elsewhere [38].

LEEDS is the first randomised controlled trial to compare buprenorphine and dihydrocodeine for opiate detoxification. Sixty people with problems of opiate dependence agreed to take part in this randomised trial. Thirty five of these people were recruited from a medical centre for the homeless. Recruitment of practitioners was problematic though recruitment of participants was not a substantial problem for practitioners committed to recruiting into the trial. This study ran on a very low budget (50% research assistant time). LEEDS illustrates how such studies, undertaken in the context of routine care, even with such potentially problematic clientele, are both possible and feasible.

One limitation of LEEDS was that it was underpowered to detect with confidence clear differences for secondary outcomes between regimens. Selecting a data collection point for the primary outcome at completion of detoxification could be seen as a limitation of the study. However, this outcome was selected after careful consideration. Ideally urine collection would be several days post-detoxification to allow for all prescribed opiates to be clear from the test. We thought, however, many users would not attend primary care after completion of detoxification simply to provide a urine test, particularly as much of the recruitment was from a homeless population who have been traditionally difficult to engage and retain in treatment services [39]. Indeed, only 23% of participants provided a final urine sample. Reasons for this are varied and multiple (Figure 1). Fourteen people did not collect their final prescription and therefore were not available at this time point to provide a urine sample. Five people never returned to the GP practice to collect any prescriptions after their first consultation with the GP or drugs worker. Additionally, 24 people failed to collect a prescription somewhere between the second and penultimate. The high numbers of people who did not provide a urine sample demonstrates the difficulty in retaining injecting drug users in treatment services. This pragmatic, low budget study only sought to record contact with GPs and drugs workers and did not have sufficient capacity to make personal contact during the detoxification period to obtain abstinence status independent of that recorded through medical contact.

The main objectives of LEEDS were to have some indication of whether one regimen was associated with better odds of completing detoxification and to test methods for larger studies. We recognize that these are limited goals but found no indication from the literature or even experts in the field that data was known for these outcomes. Of course, retention in treatment services post-detoxification is an important part of the whole treatment package offered to drug users so we also recorded the frequency of medical service utlisation by participants.

An additional limitation of the study is that we were unable to collect data on the numbers and demographics of those people who declined to participate. This was due to the busy nature of the primary care treatment setting.

Currently many drug users arrested for crimes related to drug misuse are offered a choice of legally mandated treatment (referred to by some as 'coerced' treatment) [40] or a custodial sentence. No participant in LEEDS had been legally mandated to enter treatment from the criminal justice system. Consequently, all participants expressed self motivation to undergo detoxification. Yet, regardless of which detoxification drug people were randomised to, completion rates were poor. This study suggests that even in this relatively self motivated group of people, completion rates were between only 13% and 32%. In secondary care others have reported completion rates in the range of 33% [41]. However comparisons with study retention rates from trials undertaken in secondary care should be made with caution as it is possible that participants were not equivalent in terms of motivation and self-efficacy. More evaluation of treatment effects in different health settings would therefore seem prudent and we have nearly completed a larger study in the prison setting. The results of this current study, however, are generalisable to those patients presenting for detoxification from illicit opiates in primary care. We would be less confident of generalising our findings to the residential or inpatient setting.

This trial suggests that buprenorphine may be able to deliver 20% more completion than dihydrocodeine. If completion of detoxification is associated with remaining abstinent, use of buprenorphine as an agent of opiate detoxification could be a very important step forward. Whilst clinicians prescribing the interventions were not blinded beyond the point of randomisation, the difference favouring buprenorphine could be due to increased professional input for that intervention. However there was no evidence to suggest that this was the case. Rather, there was no suggestion of a difference in GP/drug worker visits between the two groups. LEEDS was a "real-world" trial with a pragmatic rather than explanatory design. As such it randomised interventions which are used in everyday clinical practice. Previous commentators have spoken about the need to balance issues of methodological rigour (commonly referred to as internal validity) versus the feasibility of conducting trials in the real world clinical environment (commonly referred to as external validity) [42]. Inevitably there is a trade off between rigour and feasibility. For example in this case whilst the use of dummy pills was considered at the design stage, it was deemed unfeasible as it would both add to the cost of the research and also limit the independence of the trial from pharmaceutical company funding.

It could also be argued that the superior percentage of those achieving abstinence as a result of the buprenorphine intervention was because mean buprenorphine and dihydrocodeine doses were not equivalent in terms of the pharmacological opiate effect. However this is not possible to verify as the two interventions are not identical in terms of action on opiate receptors. Buprenorphine has the unusual property of being both a partial MU receptor (one of a number of opiate receptors) agonist and partial opiate antagonist whereas dihydrocodeine is a full opiate receptor agonist.

## Conclusion

Only 23% of participants completed their detoxification and gave a final urine sample. This finding suggests a high non-completion rate of primary care opiate detoxifications. A higher proportion of people randomised to buprenorphine provided a final urine sample negative for illicit opiates compared with those who received dihydrocodeine. Those allocated buprenorphine made fewer visits to the GP and drugs worker during detoxification. Additionally, more of those allocated buprenorphine were abstinent at three and six months post detoxification when compared to the dihydrocodeine group.

Currently in some treatment services in the UK the open giving of dihydrocodeine has continued despite an absence of evidence to support its clinical effectiveness. More recently, there has been a marked increase in the prescribing of buprenorphine in the UK [23]. Such an increase is in line with emerging best practice primary care guidance based primarily upon face validity for opiate detoxification. This guidance supports the use of buprenorphine but not dihydrocodeine for opiate detoxification in the primary care setting [43]. The LEEDS findings begin to support this guidance with good evidence but there is some way to go before fully confident recommendations can be made.

The findings will also have relevance to any review of current Department of Health best practice guidelines for the treatment of substance misuse [4]. Launched in 1999, they argued that GP prescribing of buprenorphine requires a greater level of clinical experience than the prescribing of dihydrocodeine. The guidelines recommend buprenorphine should only be given by a "specialist general practitioner" and dihydrocodeine by an "experienced" GP [4]. LEEDS provides little evidence to support the continued prescribing of dihydrocodeine as a first line agent for opiate detoxification by less experienced GPs in primary care, but larger, well designed, conducted and reported trials are necessary.

## Competing interests

The author(s) declare that they have no competing interests.

## Authors' contributions

NW and CA designed the study, offered project supervision and drafted the manuscript. NW was principal investigator. CA centrally managed the randomisation process and conducted statistical analysis. LS coordinated and managed the project during the latter stages, collected data and drafted the manuscript. CT conducted data collection and drafted the manuscript. VA conducted statistical analysis and commented on the results section. NO was the initial project coordinator, who assisted in the design of the study and collected data. All authors have read and approved the final manuscript.

## Funding body

Leeds Primary Care Trusts Research Consortium Priorities and Needs Funding.

## Ethics committee

Leeds Teaching Hospitals Local Research Ethics Committee.

## Pre-publication history

The pre-publication history for this paper can be accessed here:


